# Conflicting theories on addiction aetiology and the strengths and limitations of substance use disorder disease modelling

**DOI:** 10.3389/fnmol.2023.1166852

**Published:** 2023-09-06

**Authors:** Megan R. Greener, Sarah J. Storr

**Affiliations:** Biodiscovery Institute, University of Nottingham, Nottingham, United Kingdom

**Keywords:** addiction, neurobiology, behaviour, genetics, *in vivo*, *in vitro*

## Abstract

A lack of cross-disciplinary unanimity prevails within addiction research. Theories conceptualizing addiction through the three-stage brain disease model contest other theories that substance use disorder is of behavioural or genetic origin. The reverberations of this lack of consensus are noticeable throughout addiction research and within the foundations of disease modelling. The availability of methods to investigate substance use disorder are inconsistent and sometimes unrepresentative. This review discusses theories of addiction aetiology, available models for addiction research and the strengths and limitations of current practical experimental methods of study.

## 1. Introduction

Construing a universally accepted definition for addiction has been a controversial matter since deliberations arose over the first documented cases of substance abuse in the 17^th^ century (Crocq, 2007). At this time, conflicting opinions existed on whether to categorise addiction as a sin or a disease (Nathan et al., 2016). The unravelling of the cause and symptomatology of addiction has diluted morality arguments and replaced them with the concept of addiction as a brain disease (culminating in the US National Institute on Drug Abuse 1997 report) (Heilig et al., 2021). However, cross-disciplinary variation in acceptance of this definition prevails. These contrasting theories make unanimity on defining addiction a constant challenge (Hammer et al., 2013).

## 2. Theories of addiction

### 2.1. Addiction: a brain disease

The conceptual framework behind addiction as a brain disease is the reality that drugs of abuse have demonstrated the potential to alter the neural circuitry and molecular constituency of the brain (Leshner, 1997).

The transition from acute, controlled, or occasional usage to chronic drug addiction is equally paralleled with neurochemical and molecular changes. These changes can be grouped into a three-stage addiction cycle of ‘binge/intoxication’, ‘withdrawal/negative effect’ and ‘preoccupation/anticipation’ in which distinct neural remodelling is clear within stages (Koob and Volkow, 2010).

#### 2.1.1. Binge/intoxication

All drugs of abuse result in excessive dopaminergic transmission within the brain’s reward circuitry, the mesolimbic system, which originates in the ventral tegmental area (VTA) and terminates in the nucleus accumbens (NAc) (Adinoff, 2004). Aside from the mesolimbic dopaminergic system, heightened understanding of drug reward has revealed additional mechanisms influencing mesolimbic circuitry; the endogenous opioid system and the endogenous cannabinoid system (Volkow et al., 2019). Other neurotransmitters, particularly glutamate, have also been proven to augment acute drug reward (D'Souza, 2015). Thus, reward can be generalised as increased neurotransmission by substances of abuse within the mesolimbic system.

#### 2.1.2. Withdrawal/negative effect

The persistent negative state experienced during drug abstinence, colloquially known as ‘antireward’, is analogous with upregulation of the corticotropin-releasing factor (CRF) system within the extended amygdala (Koob and Le Moal, 2008). The result of hyperactive CRF-containing neurons in the amygdala is recruitment of the hypothalamic–pituitary–adrenal axis and brain stress systems (Nestler, 2005). Other stress-related neurotransmitters including noradrenaline, dynorphin, vasopressin and substance P are also overexpressed (Kwako and Koob, 2017). Activation of these stress systems correlate to the negative emotional state experienced during withdrawal (Koob, 2009). Additionally, opposing neuroadaptations to reward processes occur. This results in a reduction in dopaminergic neurotransmission and therefore diminished positive response (Feltenstein, 2008). Heightened stress and depreciated reward pathways amalgamate during this stage and present as withdrawal and resulting compulsion.

#### 2.1.3. Preoccupation/anticipation

An auxiliary of the preoccupation and anticipation stage is chronic relapse (Gutman, 2006). Further reorganisation of reward circuits and neuroplastic changes, predominantly localised to the prefrontal cortex (PFC), echo this phenomenon (Koob and Simon, 2009; Veerappa et al., 2021). Alterations of the PFC including reduction of overall grey matter volume or disruption in specific regions such as the dorsolateral PFC correlate to impaired response inhibition and salience attribution (Goldstein and Volkow, 2011). These neuroplastic changes result in over activation of ‘go’ systems and under activation of ‘stop’ systems, which are causative of habitual substance seeking and increases in impulsivity (Substance Abuse and Mental Health Services Administration (US) and Office of the Surgeon General (US), 2016). Neural network remodelling manifests physically as intense craving and a loss of self-control.

#### 2.1.4. Three-stage brain disease

Hence, specific brain alterations coincide with the accepted symptoms of addiction, namely abuse, withdrawal and relapse.

Discrepancies lie where molecular and neurochemical changes interrelate with the environmental factors of the individual (O'Brien, 1997). In response to this, behavioural manifestations (for example conditioning) can be differentially expressed in individuals despite identical substance-induced changes to neuroplasticity (Heyman, 1996). Some argue that there is heterogeneity in causation between cases, some being of brain-disease origin and others not, whilst others discount the disease theory entirely (Pickard, 2022). It is these complexities that translate to interprofessional divergence when describing or modelling addiction.

### 2.2. Addiction: a behavioural disorder

Possibly the most prominent contention to brain disease models stems from research psychologist Gene Heyman, who argues that addiction is not a disease but a voluntary choice (Heyman, 2009). Heyman’s viewpoint stemmed from psychiatric epidemiological studies and described replacement of predetermined social rules with self-centred rationalisations as the paramount explanation for addiction (Hunt, 2010).

The argument did not deny the presence of proven neurobiological alterations caused by substances of abuse but denied the capability of these changes to disturb routine decision making. This was in response to findings that many individuals recover from medically defined addiction without treatment, that many recreational drug users do not become compulsive drug users and the lack of elucidated causal link between neural adaptions and addiction (Heyman, 2013).

Other theories assert that the origin of substance use disorders is developmental learned behaviour, which nullifies the brain disease theory with claims that addictive neuroplasticity alterations mirror those seen in the development of deep habits generally (Lewis, 2017). However, common learning-behaviour theories of habitual drug-seeking describe loss of executive control over habits after neuroplasticity changes, which is harmonious with disease-rooted theories (Ostlund and Balleine, 2008).

Incentive sensitization describes the psychological form of ‘wanting’ triggered by stimulus which has been paired with neural modifications that increase incentive salience (Bechara et al., 2019). This theory differentiates ‘wanting’ a reward from ‘liking’ the reward and highlights the insubstantial contribution of neural adaptations to the latter (Berridge and Robinson, 2016).

Conditioning hypotheses, which are also rooted in drug-related environment stimuli to cause drug use, have a proven neurobiological basis linking dopaminergic transmission to drug-related cues and stress (WEISS et al., 2001). Furthermore, the different stages of condition placed preference (CPP), a leading behavioural model, have been intrinsically linked to specific neural alterations (Fattahi et al., 2023).

Models of positive and negative reinforcement (respectively drug reward and adverse withdrawal) as the origin of addiction are also supported through cognitive dysfunction mechanisms present in the three-stage addiction model (Hogarth, 2020).

Within the growing psychological movement that defines instances of gambling, over-eating, sexual intercourse and gaming as behavioural addictions, neurobiological modifications have coincided with the disorders (Chamberlain et al., 2016).

Earlier tendencies of theorists to combat disease-rooted arguments with behavioural origins entirely reveal the monumental challenge the field faces in amalgamating hypotheses and moving toward unanimous conclusions on aetiology. However, recent developments homogenise these conflicting theories to some extent through magnification of specific neuroadaptations paralleling specific behavioural tendencies. Both behavioural and neurobiological concepts are undeniable. What must be determined is the extent to which the theories are prevalent in the aetiology of addiction at different stages. The unravelling of neurobiological contributions to incentive salience are an appreciable example of this: neuroplasticity changes are relevant to ‘wanting’ but are of limited value in ‘liking’ behaviourally.

Another particularly compelling argument describes the need for promotion of ‘empirically based pluralism’ when considering alcohol dependence aetiology (Kendler, 2012). Here, it is argued that alcohol dependence is influenced by ‘difference makers’ including but not limited to molecular and systems neuroscience; social, political, or cultural influence, and genetics. Addiction, generally, can be viewed from this comprehensive vantage point, where appreciation of the contribution of multiple factors and theories are valued.

The more interconnections made between specific brain regions and specific addictive behaviours, the more conceivable it is to relate pathology to the anomalies suggested or determine a specific origin for addictive tendencies, the easier it becomes to align theories. Lack of clarity promotes inconsistencies in investigating addiction disorder, which is further augmented through theories that addiction is a genetic disease.

### 2.3. Addiction: a genetic disorder

Chronic drug usage is associated with upregulation of the cyclic adenosine 3′,5′-monophosphate (cAMP) pathway and thus dysregulation of cAMP response element–binding protein (CREB) and *fos-*like protein isoform ΔFosB (Nestler and Aghajanian, 1997). Dysregulation of these transcription factors causes alterations to gene expression (Nestler, 2004). Therefore, much research centres around the specific target genes of these transcription factors and others (Nestler, 2000).

Familial studies (twin/family/adoption) and association and linkage studies proved that many genes result in variance of addictive traits and hereditary vulnerability (Crabbe, 2002). The recent application of genome-wide association studies (GWAS) greatly advanced our understanding of addiction genetics (Hancock et al., 2018). Recently published genome scanning data has supplemented these classical genetic studies and provided researchers with reproducible chromosomal loci containing variants that could alter human addiction vulnerability (Uhl et al., 2002).

Less divergence exists between theories of addiction genetics and others. For example, application of GWAS data has been pursued to investigate the underlying neurobiology of variants and their correlation to cellular processes molecular function (Gelernter and Polimanti, 2021). Integration of genomics and neurobiology has contributed to a new ‘Genetically Informed Neurobiology of Addiction (GINA)’ model. The model not only entwines genetics and neuroscience but gives appreciation to environmental contributions of addiction origin. Hence, the GINA model is arguably the first of its kind in that it manifests all competing theories in a unified way. Further investigation of epigenetics could integrate genetic and behavioural theories further through unravelling environmentally induced alterations in gene expression (Archer et al., 2012).

### 2.4. Addiction theory

**Figure 1:**
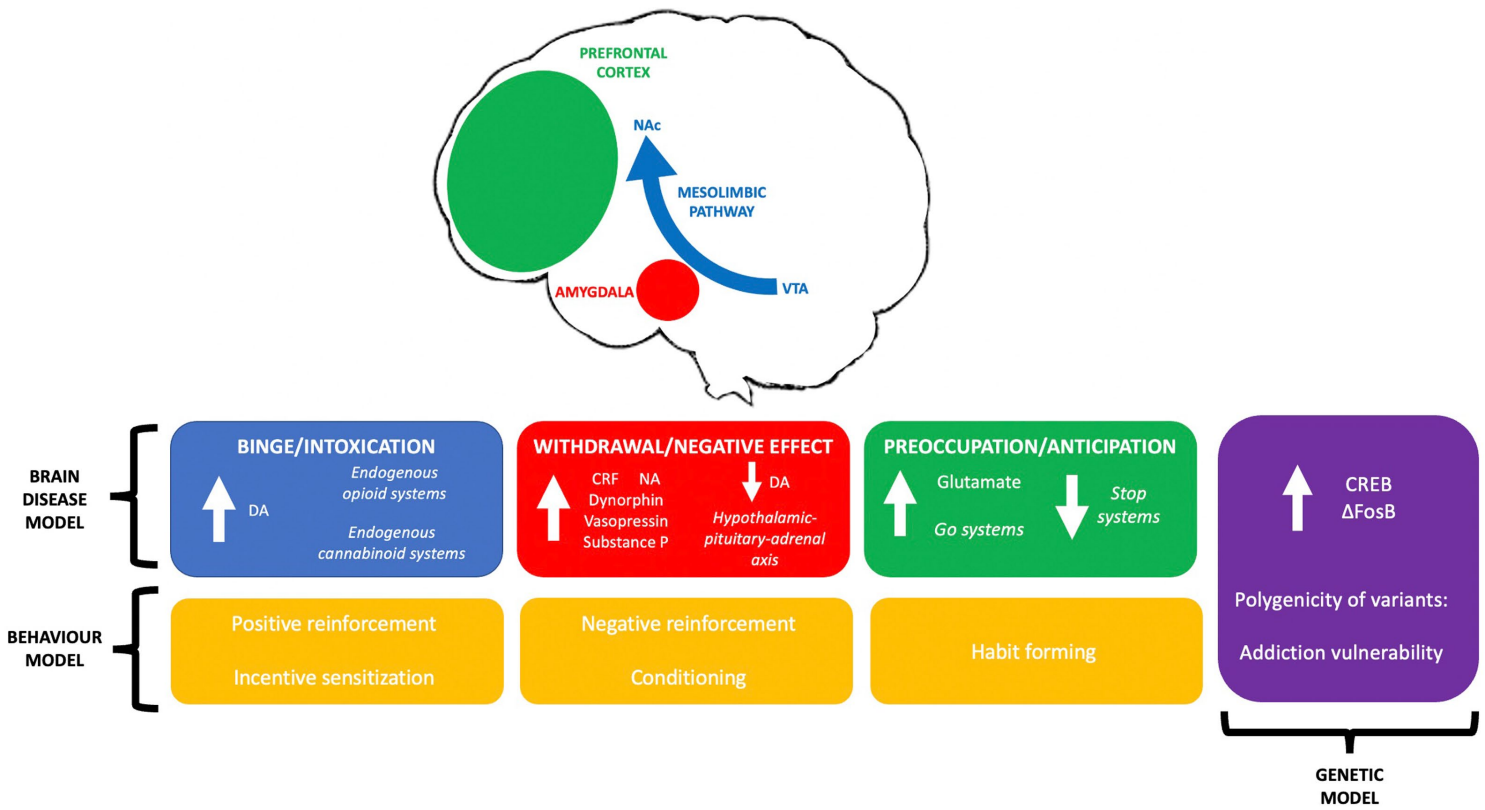
A summary of leading theories on addiction aetiology; the three-stage brain disease model, concepts in genetic modelling and behavioural theories of addiction, and their interrelationships.

A fully comprehensive literature review of all theories cannot be completed within the scope of this mini-review, but the discrepancies and concordance between multiple leading models are presented (see Figure 1).

Contrasting opinions on the causative factor of addiction between neurobiology, behaviour and genetics is commonplace in the literature across fields. Research into substance use disorder should be in lieu of the neurobiological changes associated with addiction; the interaction of these changes with the subject’s environment; the effect of these changes on behaviour; the resulting transcriptional changes, and the influence of genetics on neuroadaptation and vulnerability.

Models of addiction allowing investigation into these theories and their constituency are vital. Here, we discuss the currently available methods of research in these areas and the strengths and limitations of practical, experimental addiction modelling.

## 3. Models of addiction

### 3.1. *In vivo* addiction models

In lieu of tendencies to define addiction in degrees of abstinence, animal self-administration experiments inclusive of oral and intravenous drug-taking and intracranial self-stimulation (ICSS) have been predominant *in vivo* models (Gardner, 2000). These studies have provided valuable information on addiction sensitivities, abuse potential and neuroadaptation mechanisms (particularly within the binge and intoxication stage of the brain-disease model) (Negus and Miller, 2014). Recent trends in ICSS have highlighted the strengths of this technique in developing our comprehension of drug-reward deficits, abuse-limiting effects, addictive relativity between substances of abuse and specific neurotransmission (Kenny et al., 2018).

CPP and condition placed aversion (CPA) studies apply habituation, freedom of movement and conditioning to positive or negative stimuli to assess the motivation of reward or the negative effects of withdrawal (Cunningham et al., 2006). The advantage of these methods is the ability to investigate drug reinforcement tendencies and draw parallels between drug use and environmental cues (McKendrick and Graziane, 2020).

Within animal reinstatement experiments, the extent to which non-contingent exposure to drugs or related stimuli result in renewal of a previously drug-reinforced behaviour after extinction is measured (Shaham et al., 2003). These experiments aim to disentangle the complexities of craving and relapse.

Animal models have informed our psychological and neurobiological understanding of addiction and thus driven treatment development. In the case of alcohol use disorder (AUD), for example, results from animal experiments were translated clinically to specifically licenced medications including acamprosate and naltrexone (Spanagel, 2017).

Aside from this, animal studies possess a tendency to ineffectively translate to clinical situations. Some argue this lack of translatability is a consequence of ill-considered animal study designs, one example being the lack of alternative options to drug taking in classic self-administration studies (Ahmed, 2010). The decision to administer a substance in this case would not illustrate the decision-making process of an addicted individual, and as such is unrepresentative. Whilst animal researchers have ameliorated this to an extent through the introduction of modern methods like alternative non-drug rewards, forced versus voluntary abstinence and punishment upon reinstatement, the issue of lack of coherence between animal and human behaviour is still prevalent (Kramer et al., 2020). Some addictive criteria, such as escalation of drug use, can be efficaciously modelled in non-human animal models, whilst other addictive complexities fail in human recapitulation (Ahmed, 2011).

Reasonable debate stems from the belief that animal models fundamentally fail in replicating the complexities of an addicted human brain (Everitt et al., 2017). Whilst animal models have postdictive validity (as evident through application of postdictive models in buprenorphine, methadone, and nicotine replacement therapy development), they cannot feasibly be used as a model of initial addiction research or as a model on which to base treatment discovery without heavily considering the translational limitations (Heilig et al., 2016).

*Drosophila melanogaster* use in addiction research is practical, inexpensive, and allows investigations into genetics through utilisation of their extensive homogeneity with the human genome and their ability to engage in drug related behaviours (Devineni and Heberlein, 2010; Awofala, 2011; Kaun et al., 2012). However, similar difficulties in linking neural activity to behaviour or to what extent *Drosophila* can replicate complex human addictive characteristics prevail (Sokolowski, 2001).

Animal genetic studies such as knock out (KO) experiments have similar translational limitations, though recent progress in integrating phenotypical analysis and genomic data from addiction studies across species could improve this (Cates et al., 2019).

Human studies logically improve upon limitations of animal and *Drosophila* studies. Within human self-administration studies, the variability of addictive tendencies in patients suffering from substance use disorders can be established in research settings. Patterns of addiction characteristics such as daily drug use, bidirectionality of social factors, and drug-seeking or drug-taking behaviour depending on experimental conditions can be determined within the research setting (Venniro et al., 2016).

From human brain imaging studies, assumptions on neurobiological mechanisms of addiction can be made (Volkow et al., 2003). Modern imaging techniques including magnetic resonance spectroscopy (MRS), positron emission tomography (PET) and single photon emission computed tomography (SPECT) allow real-time observations of neuroplastic changes in response to drug use, and so are an undoubtedly useful method of addiction modelling (Fowler et al., 2007). The difficulty with these models is the legal, ethical, and practical challenge of conducting human research, especially in the setting of addiction to illegal substances. Whilst ideal in representability, human studies are beyond the scope of many research laboratories and so the lack of an elementary, practicable and translatable model for basic and mechanistic addiction research persists.

Hence, all current *in vivo* models of addiction have advantages and limitations (see Table 1). Traditionally, in many other research themes, *in vitro* models can be used to allow simplistic and workable investigations (often before progression to *in vivo* studies). The issue within the addiction research setting, and neuroscience generally, is that *in vitro* modelling of complex neurological disorders presents with complications itself.

**Table 1:** A summary of the advantages and disadvantages of platforms to model and investigate substance use disorder.

Model of addiction	Investigative theory	Advantages	Disadvantages
*In vivo:* animal models	Neurobiology, behavioural, genetics	Postdictive validation Phenotypic investigation into animal addiction neurobiology, behaviour and genetics	Low success rates translating to human clinical end goal Ethical considerations
*In vivo:* human models	Neurobiology, behavioural, genetics	Fully representative investigation of human addiction neurobiology, behaviour and genetics	High cost Ethical considerations
*In vivo:* drosophila	Neurobiology, behavioural, genetics	High homogeneity with human genome: neurobiology and genetic investigations low cost rapid and practical	Limitations associating neurobiological and behavioural activity to human
*In vitro:* human brain tissue	Neurobiology, genetics	Fully representative investigation of human addiction neurobiology and genetics	Limited accessibility
*In vitro:* SH-SY5Y	Neurobiology	Rapid and practical	Limitations in representability of neurobiological changes
*In vitro:* stem cells	Neurobiology, genetics	Fully representative investigation of human addiction neurobiology and genetics high accessibility	Variability of differentiations and therefore reproducibility due to complex protocols

### 3.2. *In vitro* addiction models

Traditional *in vitro* models of addiction have relied on human post-mortem or rodent brain tissue, often used to isolate primary neurons for culture, thus the problem of accessibility or difficulties recapitulating the human brain using animal-based models persist (Le Feber, 2019). A recent surge in novel *in vitro* preparations and techniques has provided a platform that could overcome these limitations.

Much of this research has centred around protocols allowing laboratory growth of *in vitro* neural cells that originate from embryonic stem cells (ESCs) or induced pluripotent stem cells (IPSCs) (Altimus et al., 2020). This technique provides an *in vitro* model that is innately human and manipulatable depending on cell type required or the neurobiological mechanisms under investigation (Bitar and Barry, 2020).

Within addiction research there are already multiple examples of IPSC application, for example *in vitro* investigations into cannabinoid signalling and its effect on neuronal and dopaminergic maturation (Stanslowsky et al., 2017). The method provides a platform of investigating addictive neurobiology in a setting where the results are conclusively representative of human neuroadaptations owing to their human origin.

Issues with accessing post-mortem brain tissue or reliance on human subjects for *in vivo* experiments are reduced when using stem cells, as an exponential number of experiments can be completed in a relevant neuroscientific environment from one initial human source (Goncalves and Przyborski, 2018). This is enhanced through the capability of stem cells to form three-dimensional brain organoids, as structurally accurate human brain cell types relevant to addiction can be formed *in vitro* for experimentation without reliance on post-mortem tissue (Boeshore et al., 2022). Recent application of RNA sequencing technology to complete multiplexed morphogen screens of these organoids has provided more clarity on differentiating to specific neural subtypes, allowing replication of human neuronal cells with distinct, diverse identities for experimentation (Amin et al., 2023).

Additional advantages come from comparisons of IPSCs from healthy versus addicted patients. This allows us to investigate neuroadaptations conclusively and permits investigation into genetic susceptibility or specific allele variation for investigation of both genetic and brain disease theories (Sheng et al., 2016). What is particularly promising when considering the application of stem cells to investigate neurobiology and genetics is the potential of combining this with modern sequencing capabilities as GWAS or ‘omics approaches. Having readily available human tissue for genomic investigations could promote advancement of our understanding of the complex biology behind addiction by providing a platform of practical, relevant research. Hence, novel culture techniques and sequencing technologies could provide a paradigm for experimental investigations that consider each competing theory of addiction.

Limitations of IPSC or ESC application arise from issues of reproducibility when generating the models, which is partly attributed to genomic or epigenomic variability influencing differentiation and otherwise attributed to practical discrepancies (Marchetto et al., 2011). The complexity of IPSC and ESC use is therefore an obstacle to broad scale application in addiction research, as is the reliability and reproducibility of the resulting terminally differentiated cell lines.

Refining protocols to reduce the weighting of this variation, for example through completing additional gain and loss of function experiments to exclude genetic variability, are essential, as is confirmation of the relevance of IPSCs in replicating addiction (Soldner and Jaenisch, 2017). Studies are indicating that IPSCs or ESCs are suitable *in vitro* models to study substance use disorder, giving lower weighting to the latter issue (Lieberman, 2016).

More straight forward protocols of differentiation include human neuroblastoma cell line SH-SY5Y to form dopaminergic neuronal-like cells are already used in *in vitro* molecular neuroscience (Dravid et al., 2021). These models use readily accessible immortal cell lines and there are examples of use within addiction settings, but their simplicity of use translates to lower representability compared to the ability of stem cells to reproduce specific brain cell types and the same issue of variability in differentiation transpires (Ma et al., 2015).

*In vitro* addiction modelling could allow efficient and relevant drug discovery or treatment pathways that could result in improvement of the currently substandard clinical options in addiction care, especially when combined with modern sequencing technologies (Giri and Bader, 2015). However, the models are notwithstanding limitations, and the information on neurobiology or genetics gained through *in vitro* methods must still be considered in lieu of behavioural arguments and environmental factors.

## 4. Discussion

Addiction is a uniquely challenging and complicated disorder, and so it is not entirely surprising that there are interprofessional discrepancies between definitions and theories on causation. The repercussions of this ambiguity extend throughout the field of addiction and arise even within basic disease-modelling for experimentation.

*In vivo* experiments conducted on human studies or *in vitro* experiments using human brain tissue are advantageous to addiction research, allowing insights into human behaviour and the neurobiological changes associated with substance use disorder. However, limitations exist due to lack of availability, practicalities, and the financial burden of human studies.

The use of IPSCs or ESCs to recapitulate the human brain *in vitro* could improve upon some of the current limitations of addiction modelling and may provide a platform for practical and reproducible addiction research through forming structurally, biologically, and genetically relevant *in vitro* environments. Combining this method with contemporary sequencing techniques permits thorough exploration of the latter, and through epigenetic approaches could reduce disparities between these models and behavioural theories of addiction.

Appreciation of all theories of addiction aetiology and their coordination with each other is vital to improving our understanding of substance use disorder and translating this to effective treatment pathways. The lack of a practical, representative model for addiction studies is not only an obstacle to advancing this understanding, but likely a direct result of this lack of clarity.

Modern techniques have the potential to overcome the shortcomings of previously utilised methods by providing researchers with innately human, practical, and manipulatable addiction models. This could be instrumental to effective drug discovery for substance use disorder and has the potential to revolutionise the field. However, acceptance of concurrent theories in addiction aetiology must still precede a universally accepted experimental model.

## Author contributions

MG: original draught preparation. SS: review and editing. All authors have read and agreed to the published version of the manuscript.

## Funding

This work was funded in whole, or in part, by the Wellcome Trust [RS3684]. For the purpose of open access, the author has applied a CC BY public copyright licence to any Author Accepted Manuscript version arising from this submission.

## Conflict of interest

The authors declare that the research was conducted in the absence of any commercial or financial relationships that could be construed as a potential conflict of interest.

## Publisher’s note

All claims expressed in this article are solely those of the authors and do not necessarily represent those of their affiliated organizations, or those of the publisher, the editors and the reviewers. Any product that may be evaluated in this article, or claim that may be made by its manufacturer, is not guaranteed or endorsed by the publisher.

## References

[ref1] Adinoff B. (2004). Neurobiologic processes in drug reward and addiction. Harv. Rev. Psychiatry 12, 305–320. doi: 10.1080/10673220490910844, PMID: 15764467 PMC1920543

[ref2] Ahmed S. H. (2010). Validation crisis in animal models of drug addiction: beyond nondisordered drug use toward drug addiction. Neurosci. Biobehav. Rev. 35, 172–184. doi: 10.1016/j.neubiorev.2010.04.005, PMID: 20417231

[ref3] Ahmed S. H. (2011). “Escalation of drug use” in Animal models of drug addiction. ed. Olmstead M. C. (Totowa, NJ: Humana Press)

[ref4] Altimus C. M. Marlin B. J. Charalambakis N. E. Colón-Rodriquez A. Glover E. J. Izbicki P.. (2020). The next 50 years of neuroscience. J. Neurosci. 40, 101–106. doi: 10.1523/jneurosci.0744-19.2019, PMID: 31896564 PMC6939479

[ref5] Amin N. D. Kelley K. W. Hao J. Miura Y. Narazaki G. Li T.. (2023). Generating human neural diversity with a multiplexed morphogen screen in organoids. bio Rxiv. 2023–05. doi: 10.1101/2023.05.31.541819 PMC12302009 39642864

[ref6] Archer T. Oscar-Berman M. Blum K. Gold M. (2012). Neurogenetics and epigenetics in impulsive behaviour: impact on reward circuitry. J. Genet. Syndr. Gene. Ther. 3:1000115. doi: 10.4172/2157-7412.1000115, PMID: 23264884 PMC3526005

[ref7] Awofala A. A. (2011). Genetic approaches to alcohol addiction: gene expression studies and recent candidates from Drosophila. Invertebr. Neurosci. 11, 1–7. doi: 10.1007/s10158-010-0113-y, PMID: 21153676

[ref8] Bechara A. Berridge K. C. Bickel W. K. Morón J. A. Williams S. B. Stein J. S. (2019). A neurobehavioral approach to addiction: implications for the opioid epidemic and the psychology of addiction. Psychol. Sci. Public Interest 20, 96–127. doi: 10.1177/1529100619860513, PMID: 31591935 PMC7001788

[ref9] Berridge K. C. Robinson T. E. (2016). Liking, wanting, and the incentive-sensitization theory of addiction. Am. Psychol. 71, 670–679. doi: 10.1037/amp0000059, PMID: 27977239 PMC5171207

[ref10] Bitar M. Barry G. (2020). Building a human brain for research. Front. Mol. Neurosci. 13:22. doi: 10.3389/fnmol.2020.00022, PMID: 32132903 PMC7040093

[ref11] Boeshore K. L. Lee C.-T. Freed W. J. (2022). “Chapter 6-Applications of human induced pluripotent stem cell and human embryonic stem cell models for substance use disorders: addiction and neurodevelopmental toxicity” in Novel concepts in iPSC disease modeling. ed. Birbrair A. (Academic Press), 153–177. doi: 10.1016/B978-0-12-823882-0.00006-0

[ref12] Cates H. M. Benca-Bachman C. E. de Guglielmo G. Schoenrock S. A. Shu C. Kallupi M. (2019). National Institute on Drug Abuse genomics consortium white paper: coordinating efforts between human and animal addiction studies. Genes Brain Behav. 18:e12577. doi: 10.1111/gbb.12577, PMID: 31012252 PMC7891887

[ref13] Chamberlain S. R. Lochner C. Stein D. J. Goudriaan A. E. van Holst R. J. Zohar J.. (2016). Behavioural addiction–a rising tide? Eur. Neuropsychopharmacol. 26, 841–855. doi: 10.1016/j.euroneuro.2015.08.013 26585600

[ref14] Crabbe J. C. (2002). Genetic contributions to addiction. Annu. Rev. Psychol. 53, 435–462. doi: 10.1146/annurev.psych.53.100901.135142 11752492

[ref15] Crocq M.-A. (2007). Historical and cultural aspects of man's relationship with addictive drugs. Dialogues Clin. Neurosci. 9, 355–361. doi: 10.31887/DCNS.2007.9.4/macrocq, PMID: 18286796 PMC3202501

[ref16] Cunningham C. L. Gremel C. M. Groblewski P. A. (2006). Drug-induced conditioned place preference and aversion in mice. Nat. Protoc. 1, 1662–1670. doi: 10.1038/nprot.2006.279, PMID: 17487149

[ref17] Devineni A. V. Heberlein U. (2010). Addiction-like behavior in Drosophila. Commun. Integr. Biol. 3, 357–359. doi: 10.4161/cib.3.4.11885, PMID: 20798826 PMC2928318

[ref18] Dravid A. Raos B. Svirskis D. O’Carroll S. J. (2021). Optimised techniques for highthroughput screening of differentiated SH-SY5Y cells and application for neurite outgrowth assays. Sci. Rep. 11:23935. doi: 10.1038/s41598-021-03442-1, PMID: 34907283 PMC8671469

[ref19] D'Souza M. S. (2015). Glutamatergic transmission in drug reward: implications for drug addiction. Front. Neurosci. 9:404. doi: 10.3389/fnins.2015.00404, PMID: 26594139 PMC4633516

[ref20] Everitt B. J. Giuliano C. Belin D. (2017). Addictive behaviour in experimental animals: prospects for translation. Philos. Trans. R. Soc. Lond. Ser. B Biol. Sci. 373:20170027. doi: 10.1098/rstb.2017.0027, PMID: 29352026 PMC5790825

[ref21] Fattahi M. Modaberi S. Eskandari K. Haghparast A. (2023). A systematic review of the local field potential adaptations during conditioned place preference task in preclinical studies. Synapse 77:e22277. doi: 10.1002/syn.22277, PMID: 37279942

[ref22] Feltenstein M. W. (2008). The neurocircuitry of addiction: an overview. Br. J. Pharmacol. 154, 261–274. doi: 10.1038/bjp.2008.51, PMID: 18311189 PMC2442446

[ref23] Fowler J. S. Volkow N. D. Kassed C. A. Chang L. (2007). Imaging the addicted human brain. Sci. Pract. Perspect. 3, 4–16. doi: 10.1151/spp07324, PMID: 17514067 PMC2851068

[ref24] Gardner E. L. (2000). What we have learned about addiction from animal models of drug self-administration. Am. J. Addict. 9, 285–313. doi: 10.1080/10550490075004735 11155784

[ref25] Gelernter J. Polimanti R. (2021). Genetics of substance use disorders in the era of big data. Nat. Rev. Genet. 22, 712–729. doi: 10.1038/s41576-021-00377-1, PMID: 34211176 PMC9210391

[ref26] Giri S. Bader A. (2015). A low-cost, high-quality new drug discovery process using patient-derived induced pluripotent stem cells. Drug Discov. Today 20, 37–49. doi: 10.1016/j.drudis.2014.10.011 25448756

[ref27] Goldstein R. Z. Volkow N. D. (2011). Dysfunction of the prefrontal cortex in addiction: neuroimaging findings and clinical implications. Nat. Rev. Neurosci. 12, 652–669. doi: 10.1038/nrn3119, PMID: 22011681 PMC3462342

[ref28] Goncalves K. Przyborski S. (2018). The utility of stem cells for neural regeneration. Brain Neurosci. Adv. 2:2398212818818071. doi: 10.1177/2398212818818071 32166173 PMC7058206

[ref29] Gutman S. A. (2006). Why addiction has a chronic, relapsing course. The neurobiology of addiction. Occup. Ther. Ment. Health 22, 1–29. doi: 10.1300/J004v22n02_01

[ref30] Hammer R. Dingel M. Ostergren J. Partridge B. McCormick J. Koenig B. A. (2013). Addiction: current criticism of the brain disease paradigm. AJOB Neurosci. 4, 27–32. doi: 10.1080/21507740.2013.796328, PMID: 24693488 PMC3969751

[ref31] Hancock D. B. Markunas C. A. Bierut L. J. Johnson E. O. (2018). Human genetics of addiction: new insights and future directions. Curr. Psychiatry Rep. 20:8. doi: 10.1007/s11920-018-0873-3, PMID: 29504045 PMC5983372

[ref32] Heilig M. Epstein D. H. Nader M. A. Shaham Y. (2016). Time to connect: bringing social context into addiction neuroscience. Nat. Rev. Neurosci. 17, 592–599. doi: 10.1038/nrn.2016.67, PMID: 27277868 PMC5523661

[ref33] Heilig M. Mac Killop J. Martinez D.. (2021). Addiction as a brain disease revised: why it still matters, and the need for consilience. Neuropsychopharmacology 46, 1715–1723. doi: 10.1038/s41386-020-00950-y, PMID: 33619327 PMC8357831

[ref34] Heyman G. M. (1996). Resolving the contradictions of addiction. Behav. Brain Sci. 19, 561–574. doi: 10.1017/S0140525X00042990

[ref35] Heyman GM. (2009). Addiction: a disorder of choice. Cambridge Harvard University Press.

[ref36] Heyman G. (2013). Addiction and choice: theory and new data. Front. Psych. 4:31. doi: 10.3389/fpsyt.2013.00031 PMC3644798 23653607

[ref37] Hogarth L. (2020). Addiction is driven by excessive goal-directed drug choice under negative affect: translational critique of habit and compulsion theory. Neuropsychopharmacology 45, 720–735. doi: 10.1038/s41386-020-0600-8, PMID: 31905368 PMC7265389

[ref38] Hunt D. W. (2010). A review of addiction: a disorder of choice. J. Addict. Dis. 29, 412–413. doi: 10.1080/10550887.2010.490471

[ref39] Kaun K. R. Devineni A. V. Heberlein U. (2012). *Drosophila melanogaster* as a model to study drug addiction. Hum. Genet. 131, 959–975. doi: 10.1007/s00439-012-1146-6, PMID: 22350798 PMC3351628

[ref40] Kendler K. S. (2012). The dappled nature of causes of psychiatric illness: replacing the organic-functional/hardware-software dichotomy with empirically based pluralism. Mol. Psychiatry 17, 377–388. doi: 10.1038/mp.2011.182 22230881 PMC3312951

[ref41] Kenny P. J. Hoyer D. Koob G. F. (2018). Animal models of addiction and neuropsychiatric disorders and their role in drug discovery: honoring the legacy of Athina Markou. Biol. Psychiatry. 83, 940–946. doi: 10.1016/j.biopsych.2018.02.009, PMID: 29602521

[ref42] Koob G. F. (2009). Brain stress systems in the amygdala and addiction. Brain Res. 1293, 61–75. doi: 10.1016/j.brainres.2009.03.038, PMID: 19332030 PMC2774745

[ref43] Koob G. F. Le Moal M. (2008). Addiction and the brain antireward system. Annu. Rev. Psychol. 59, 29–53. doi: 10.1146/annurev.psych.59.103006.093548 18154498

[ref44] Koob G. F. Simon E. J. (2009). The neurobiology of addiction: where we have been and where we are going. J. Drug Issues 39, 115–132. doi: 10.1177/002204260903900110, PMID: 20622969 PMC2901107

[ref45] Koob G. F. Volkow N. D. (2010). Neurocircuitry of addiction. Neuropsychopharmacology 35, 217–238. doi: 10.1038/npp.2009.110, PMID: 19710631 PMC2805560

[ref46] Kramer J. Dick D. M. King A. Ray L. A. Sher K. J. Vena A.. (2020). Mechanisms of alcohol addiction: bridging human and animal studies. Alcohol Alcohol. 55, 603–607. doi: 10.1093/alcalc/agaa068, PMID: 32781467 PMC7576503

[ref47] Kwako L. E. Koob G. F. (2017). Neuroclinical framework for the role of stress in addiction. Chronic Stress. 1:247054701769814. doi: 10.1177/2470547017698140, PMID: 28653044 PMC5482275

[ref48] Le Feber J. (2019). In vitro models of brain disorders. Adv Neurobiol. 22, 19–49. doi: 10.1007/978-3-030-11135-9_2 31073931

[ref49] Leshner A. I. (1997). Addiction is a brain disease, and it matters. Science 278, 45–47. doi: 10.1126/science.278.5335.45 9311924

[ref50] Lewis M. (2017). Addiction and the brain: development, not disease. Neuroethics 10, 7–18. doi: 10.1007/s12152-016-9293-4, PMID: 28725282 PMC5486526

[ref51] Lieberman R. (2016). Utilizing human induced pluripotent stem cells in the study of alcohol use disorder Doctoral Dissertations, 1035.

[ref53] Ma J. Yuan X. Qu H. Zhang J. Wang D. Sun X.. (2015). The role of reactive oxygen species in morphine addiction of SH-SY5Y cells. Life Sci. 124, 128–135. doi: 10.1016/j.lfs.2015.01.003, PMID: 25623851

[ref54] Marchetto M. C. Brennand K. J. Boyer L. F. Gage F. H. (2011). Induced pluripotent stem cells (iPSCs) and neurological disease modeling: progress and promises. Hum. Mol. Genet. 20, R109–R115. doi: 10.1093/hmg/ddr336 21828073 PMC4447776

[ref55] McKendrick G. Graziane N. M. (2020). Drug-induced conditioned place preference and its practical use in substance use disorder research. Front. Behav. Neurosci. 14:582147. doi: 10.3389/fnbeh.2020.582147, PMID: 33132862 PMC7550834

[ref56] Nathan P. E. Conrad M. Skinstad A. H. (2016). History of the concept of addiction. Annu. Rev. Clin. Psychol. 12, 29–51. doi: 10.1146/annurev-clinpsy-021815-093546 26565120

[ref57] Negus S. S. Miller L. L. (2014). Intracranial self-stimulation to evaluate abuse potential of drugs. Pharmacol. Rev. 66, 869–917. doi: 10.1124/pr.112.007419, PMID: 24973197 PMC4081730

[ref58] Nestler E. J. (2000). Genes and addiction. Nat. Genet. 26, 277–281. doi: 10.1038/8157054 11062465

[ref59] Nestler E. J. (2004). Molecular mechanisms of drug addiction. Neuropharmacology 47, 24–32. doi: 10.1016/j.neuropharm.2004.06.031 15464123

[ref60] Nestler E. J. (2005). Is there a common molecular pathway for addiction? Nat. Neurosci. 8, 1445–1449. doi: 10.1038/nn1578 16251986

[ref61] Nestler E. J. Aghajanian G. K. (1997). Molecular and cellular basis of addiction. Science 278, 58–63. doi: 10.1126/science.278.5335.58 9311927

[ref62] O'Brien C. P. (1997). A range of research-based pharmacotherapies for addiction. Science 278, 66–70. doi: 10.1126/science.278.5335.66 9311929

[ref63] Ostlund S. B. Balleine B. W. (2008). On habits and addiction: an associative analysis of compulsive drug seeking. Drug Discov. Today Dis. Models. 5, 235–245. doi: 10.1016/j.ddmod.2009.07.004, PMID: 20582332 PMC2891067

[ref64] Pickard H. (2022). Is addiction a brain disease? A plea for agnosticism and heterogeneity. Psychopharmacology 239, 993–1007. doi: 10.1007/s00213-021-06013-4, PMID: 34825924

[ref65] Shaham Y. Shalev U. Lu L. de Wit H. Stewart J. (2003). The reinstatement model of drug relapse: history, methodology and major findings. Psychopharmacology 168, 3–20. doi: 10.1007/s00213-0021224-x, PMID: 12402102

[ref66] Sheng Y. Filichia E. Shick E. Preston K. L. Phillips K. A. Cooperman L.. (2016). Using iPSC-derived human DA neurons from opioiddependent subjects to study dopamine dynamics. Brain Behav. 6:e00491. doi: 10.1002/brb3.491 27547496 PMC4884574

[ref68] Sokolowski M. B. (2001). Drosophila: genetics meets behaviour. Nat. Rev. Genet. 2, 879–890. doi: 10.1038/35098592 11715043

[ref69] Soldner F. Jaenisch R. (2017). “In vitro modeling of complex neurological diseases” in Genome editing in neurosciences. eds. Jaenisch R. Zhang F. Gage F. (Cham: Springer International Publishing), 1–19. doi: 10.1007/978-3-319-60192-2_1 31314444

[ref70] Spanagel R. (2017). Animal models of addiction. Dialogues Clin. Neurosci. 19, 247–258. doi: 10.31887/DCNS.2017.19.3/rspanagel, PMID: 29302222 PMC5741108

[ref71] Stanslowsky N. Jahn K. Venneri A. Naujock M. Haase A. Martin U.. (2017). Functional effects of cannabinoids during dopaminergic specification of human neural precursors derived from induced pluripotent stem cells. Addict. Biol. 22, 1329–1342. doi: 10.1111/adb.12394, PMID: 27027565

[ref72] Substance Abuse and Mental Health Services Administration (US) and Office of the Surgeon General (US). (2016). Facing addiction in America: the surgeon general’s report on alcohol, drugs, and health. US Department of Health and Human Services: Washington, DC. 28252892

[ref73] Uhl G. R. Liu Q.-R. Naiman D. (2002). Substance abuse vulnerability loci: converging genome scanning data. Trends Genet. 18, 420–425. doi: 10.1016/S01689525(02)02719-1, PMID: 12142011

[ref74] Veerappa A. Pendyala G. Guda C. (2021). A systems omics-based approach to decode substance use disorders and neuroadaptations. Neurosci. Biobehav. Rev. 130, 61–80. doi: 10.1016/j.neubiorev.2021.08.016, PMID: 34411560 PMC8511293

[ref75] Venniro M. Caprioli D. Shaham Y. (2016). Animal models of drug relapse and craving: from drug priming-induced reinstatement to incubation of craving after voluntary abstinence. Prog. Brain. Res., 25–52. doi: 10.1016/bs.pbr.2015.08.004 26822352

[ref76] Volkow N. D. Fowler J. S. Wang G. J. (2003). The addicted human brain: insights from imaging studies. J. Clin. Invest. 111, 1444–1451. doi: 10.1172/jci18533, PMID: 12750391 PMC155054

[ref77] Volkow N. D. Michaelides M. Baler R. (2019). The neuroscience of drug reward and addiction. Physiol. Rev. 99, 2115–2140. doi: 10.1152/physrev.00014.2018, PMID: 31507244 PMC6890985

[ref78] Weiss F. Ciccocioppo R. Parsons L. H. Katner S. Liu X. Zorrilla E. P.. (2001). Compulsive drug-seeking behavior and relapse. Ann. N. Y. Acad. Sci. 937, 1–26. doi: 10.1111/j.1749-6632.2001.tb03556.x 11458532

